# Patients’ perception of using telehealth for type 2 diabetes management: a phenomenological study

**DOI:** 10.1186/s12913-018-3353-x

**Published:** 2018-07-13

**Authors:** Puikwan A. Lee, Geva Greenfield, Yannis Pappas

**Affiliations:** 1 Department of Primary Care and Public Health, School of Public Health, Imperial College London, London, UK; 2 Institute for Health Research, University of Bedfordshire, Luton, UK

**Keywords:** Telehealth, Type 2 diabetes mellitus, Phenomenology, Qualitative research, Self-management

## Abstract

**Background:**

There is a growing body of evidence that supports the uses of telehealth to monitor and manage people with diabetes at a distance. Despite this, the uptake of telehealth has been low. The objective of this study is to explore patients’ perceptions of using telehealth for type 2 diabetes management.

**Methods:**

Semi-structured interviews were undertaken with 10 patients from the NHS Newham area in London, UK. Data were collected using recorded semi-structured interviews. The interviews were transcribed verbatim and the analysis was guided by the phenomenological analysis approach.

**Results:**

We identified three main themes for facilitating positive patient experience or acceptance of telehealth and these included: technology consideration, service perceptions and empowerment. All patients asserted that they were pleased with the technology and many also proclaimed that they could not see themselves being without it. Moreover, very few negative views were reported with respect to the use of telehealth.

**Conclusion:**

The patients’ perceived telehealth as a potential to enhance their quality of life, allow them to live independently at home as well as help them take and be in more control over their own health state. The findings of this study therefore supports the use of telehealth for the routine care of people with type 2 diabetes. However, one must interpret the results with caution due to limitations identified in the sample.

## Background

Diabetes is a serious, chronic condition that is recognised as an important cause of premature death and disability worldwide. In particular, the prevalence of type 2 diabetes is emerging as one of the greatest global public health challenges in twenty-first century [1]. In the UK, the National Health Service (NHS) spends around £9.8 billion a year on diabetes. Most of this cost (80%) is spent on treating complications alone as a result of poorly controlled diabetes, of which many are preventable [2], such as blindness, kidney failure, heart attacks, strokes and amputations. Diabetes UK warned that “diabetes is threatening to bankrupt the NHS after a 60% rise in cases in the past 10 years”. The cost of treating diabetes complications is also expected to almost double by 2035/6 if no actions are taken to prevent these complications [3]. The urgent need for improvements in effective management of diabetes and preventing its complications is therefore evident.

There is a growing body of evidence that supports the uses of innovative technologies, such as telehealth, to monitor and manage people with diabetes at a distance and as frequently as it is needed [4–6]. However, despite evidence from randomised controlled trials suggesting that telehealth has the potential to improve diabetes health [6–9] the uptake of telehealth has been slow [10, 11]. There are evidence available on patients’ satisfaction and perceptions of telehealth that have contributed to our knowledge and understanding of the factors that may influence the uptake of telehealth by patients [12–14]. However, majority of these studies have taken a quantitative approach, which is limited by the ability to probe answers. In addition, the technological approaches explored are not necessarily telehealth, which is generally defined as involving the remote exchange of medical data between patients and healthcare professionals as well as the need for active participation by patients to measure vital signs using telehealth technologies [15–17]. There is also often no explanation provided in studies as to what theoretical framework was used (if there was one) to guide the entire research, making a true interpretation of findings difficult. Furthermore, little attempt has been made on investigating its impact on individual chronic diseases such as type 2 diabetes. There is therefore a paucity of data using a theoretical framework examining patients’ perceptions of telehealth for type 2 diabetes management.

The aim of this study was therefore to use semi-structured interviews to explore patients’ perceptions of using telehealth for type 2 diabetes management, using a descriptive phenomenological approach to guide the collection and interpretation of data.

For the purpose of this study, telehealth technology was defined according to the world’s largest randomised controlled trial of telehealth and telecare, the Whole System Demonstrator (WSD) programme: “*Electronic sensors or equipment that monitors vital health signs remotely, e.g. in your own home or while on the move. These readings are automatically transmitted to an appropriately trained person who can monitor the health vital signs and make decisions about potential interventions in real time, without the patient needing to attend a clinic”.* The WSD programme was funded by the Department of Health to find out how technology could help people manage their long-term health conditions including diabetes, heart failure and/or chronic obstructive pulmonary disease, while maintaining their independence [16].

## Methods

### Procedure

This study recruited participants from one of the WSD programme’s three research sites, the NHS Newham. After the end of the WSD trials, the legacy of the WSD in Newham has helped transform the East London NHS Foundation Trusts community based services to establish the Newham telehealth team. Potential participants for the interview study were identified using the Newham telehealth team’s patient database. Patients who met all of the following study inclusion criteria were initially approached with an informal invitation telephone call made by the principal researcher (PAL): i) adults ≥18 years of age with a formal diagnosis of type 2 diabetes; ii) have received or are receiving telehealth care for type 2 diabetes; iii) speak English fluently and iv) able to provide informed consent to take part in the study. The initial telephone call consisted of a formal introduction of the researcher, followed by a brief description and purpose of the study to see who would be interested in participating. If the potential patients expressed an interest in the study, a formal invitation letter was then sent out to them. The letter consisted of a participant information sheet explaining, in more detail, the purpose of the research and what would be expected of them. If patients had any questions or concerns about the study and/or were interested in taking part, they were invited to contact the researcher to discuss their questions/concerns and/or arrange a mutually agreed location and time for the interview. If no response to the invitation letter was received after 2 weeks, a follow-up telephone call was made.

On the day of the interview, before each of the interviews took place, each participant was first asked to read and sign a consent form. A brief introduction to the research topic of interest and a detailed description of what the interview entailed were given to each participant, including the use of a digital recorder during the interview. Interviews were conducted once only and the length of each interview was approximately 45 min. Following the interviews, the semi-structured interviews were transcribed for analysis. The interviews took place in patients’ preferred location, which was in their homes.

### Constructing the semi-structured interview

Topic guides were used to guide the interview process. These were developed in advance by the principal researcher (PAL) and were based on knowledge gaps identified in the current literature. The topics that were covered in the interview consisted of investigating (a) patients’ experience of having to interact with their healthcare team using technology as the mediating mechanism from home or while on the move (b) if patients had experienced anything that is particularly helpful or positive about using technology as the mediating mechanism in communicating with and receiving care from their healthcare team, (c) the difficulties and challenges associated with being unwell and using technology as the mediating mechanism in communicating with and receiving care from their healthcare team and [4] the experiences identified as being some of the most significant changes that had occurred since the use telehealth to manage their condition.

### Phenomenological method

The method of analysis used in this study was Giorgi’s descriptive phenomenological method. It consists of five main steps [18], designed to condense and highlight the themes and components that make up the meaning units of the phenomenon in question.

The first step in the data analysis was for the researcher to assume the phenomenological attitude. The second step required the researcher to read and re-read the entire description of the person’s account to develop a sense of the whole experience and become familiar with the data (18). The third step involved identifying units of general meaning that captured and described the essence of participant’s lived experience of the phenomenon of interest. The fourth step required the researcher to carefully transform each unit of general meaning originally expressed in the participants’ own words, into statements that expressed its essential psychological meaning, without changing the meaning content. The fifth and final step in the analysis involved synthesising the insights into a descriptive structure of the meaning of the experience.

## Results

### Patient characteristics

Ten out of 35 patients who were approached volunteered to participate in this study, including six of Caucasian, three of African and one of Asian ethnicities and majority of the patients were female (*n* = 8). Their ages ranged between 49 and 77 years, with a mean age of 62.6 years. Of the ten patients, four lived alone, three lived with a partner and three lived with their partner and children. Only one patient, who lived alone, had a home carer who cared for her because she has had both her legs amputated due to her diabetes condition. All of the other patients received support from their partners and/or other family members. The time since the diabetes diagnosis ranged from four to 33 years, with a mean time of 15.4 years and two patients also reported to be insulin-requiring. In addition to having diabetes, all of the patients suffered from one or more of the following chronic conditions: heart disease, hypertension, asthma, chronic obstructive pulmonary disease (COPD) and kidney problems. All of the patients used telehealth to monitor their blood glucose, blood pressure and weight and the duration of telehealth usages ranged from 1.5 years to 3.5 years.

### Overview of themes

We identified 3 broad themes that were common across patients with regards to their perception of using telehealth in managing type 2 diabetes. These themes were further subdivided into sub-themes as illustrated in Table 1.

**Table 1:** Analytical themes and sub-themes

Themes	Sub-themes
Technology consideration	• Initial perception of using technology for self-management • Telehealth usability concerns
Service perceptions	• Sense of security and comfort • Easy and convenience access to healthcare services • Privacy concerns • Continuity of care
Empowerment	• Patient education • Supporting self-care with telehealth system’s health trend analysis

### Theme 1: Technology considerations

#### Initial perception of using technology for self-management

Majority of the interviewed patients had no issues or concerns when telehealth technology was introduced to them because they regarded technology to be part of today’s modern world and that it is something you just have to keep up with. One patient specifically noted: “*No, I don’t mind that. It’s all modern things isn’t it now? You’ve got to accept it, really….”*. Those who expressed initial concerns; these were generally related to their lack of familiarity with technology and also the ability to use the equipment correctly. However, any initial concerns were quickly dismissed after a demonstration and explanation on its use was provided. One patient noted: “*I was a bit anxious because I didn’t know what it involves, and as I said I’m not a very technical person at all. So initial thing I was a bit anxious as to whether I’ll be able to use them or not. But, as I said, when the engineer went through with me, and then the nurses came, it was quite easy….”.*

#### Telehealth usability concerns

Nearly all patients commented on the simplicity of the use of telehealth technology and remarked that even a non-technical person would find it fairly easy to operate. One patient specifically noted: “*Because I am not that sort of person to go on any computers or anything. I say it is so easy to do once you get into the swing of it, ever so easy to do…. It’s not complicated at all. All you’ve got to do is just switch it on and start pressing the buttons and they come up they tell you, it tells you what to do…”.* However, despite majority of the patients found telehealth easy to operate, a few patients identified a number of issues with some of the alerts and questions asked on the system. One patient described her confusion with the timing of the alerts on the telehealth system: “*I do find it odd… If I say I do it [the recordings] today, they start flashing me at half past two in the morning with their questions about it... It [the light on the telehealth system] flashes in my bleeding eyes, it doesn’t matter what I put to block it, it still reflects off the walls…”*. Others described their annoyance with repeating alerts that had already been addressed: *“Sometimes, it would flash all through the day for the same question, which I’ve already answered the first time around…. if it was a different question, I would not mind. But when it is the same question, you get about 6 or 8 times…”*. Another patient also noted, “*It is quite a big penny size white light that flashes on the north. If you imagine you are sitting there watching T.V. and that’s constantly glaring…”.*

In addition to some alert issues, one patient who had multiple health conditions also described her frustration with the “one answer per question” restriction on the telehealth system. She expressed that she did not feel that the questions asked were appropriately tailored to her diagnoses and that it limited her ability to provide a response that fully reflected her health status: *“They only ask for one answer and you’ll think, I want to say this, this and this but you can’t because they only asked for one….I’ve got a lot of problems and I know that so I can’t rightfully answer the questions…”.*

### Theme 2: Service perceptions

#### “Sense of security and comfort”

Patients highly valued the telehealth team for always being there for them 24/7, monitoring their health and providing advice for them, even during weekends, when healthcare services are limited. One patient even described telehealth as the “Big Brother” who is always watching them at a distance. Patients also mentioned that if they had any concerns with their health, they know that telehealth is always only a phone call away. This perception of having the telehealth team monitoring and caring for them remotely around the clock provided patients with a great sense of security and comfort. One patient described: *I’m less anxious because I know someone’s always there on the other end… especially weekends, there’s no doctors, is there? They’re [Telehealth team] calling you up all the time, and they’re telling you what to do all the time….* Another patient also expressed her appreciation of the telehealth team and service: “*You can’t pop into your local diabetic clinic every week, there’re just too many people with diabetes now. But you can speak to someone in Telehealth every week; you can speak to someone in Telehealth every day if you wanted to. When I’ve had questions not really about Telehealth system but about diabetes in general, the people on the other end of the call I found them to be very knowledgeable. They’re not just call centre people, they’re healthcare professionals. I have found them very knowledgeable and very helpful. The fact that I haven’t had any emergency hospital visit is because they’re there”.*

Some patients also found the telehealth system at home useful in helping them confirm their health status, particularly on days when they felt a bit “under the weather” and were not sure whether they needed to seek medical attention: *“You have got everything that can help you [with telehealth]…. If your blood pressure was high, you’re going to know, wouldn’t you? If your diabetes was high, you know, what is going on? Your breathing, you know it sank seriously… so you’ve got to get to the doctor’s at the hospital! If I didn’t have that [telehealth], I wouldn’t know that…. I would be sitting there thinking of, “Should I go? Should I not go? Will I go at the hospital? Oh no, I’ll be all right tomorrow.” But, having that is telling me my ratings are high. So I’ll know for sure…I’m not playing a bet…. I’m not imagining things….”*.

Patients also particularly valued the reduced need to travel to see a doctor or wait for a doctor’s appointment to have their health status confirmed as they can now do it themselves via telehealth in the comfort of their own homes. One patient explained: *“I’m glad because I don’t have to keep on calling a doctor and saying, “Can I see you please because I think my blood pressure is high, or my sugar,” because I’ve got things here to check. It is up on the screen….as I’m saying, it’s [telehealth] very good it reassures me….”.* One patient also described that telehealth has likely helped her reduce the need for using the emergency/ hospital services: *“Very good because it helps me in the respect that when I’m feeling a bit under, I quickly do my blood, and it tells me whether I’m higher or I’m lower. And especially when I’m low, because then I know that I’ve got to run and get some stuff down me, some sugar or whatever…. it [telehealth] has helped me on so many occasions... If I have had to wait to go round to go see a doctor with an appointment…. “Boom”!”.*

The majority of the patients also remarked that whenever their readings were outside of the normal range, the telehealth team has always been very quick at contacting them, either by sending a message through the telehealth system, which then showed up on their TV screen, or ringing them to ensure that they are well. One patient noted, “*I’ve just done my pressure now and it was sky high, they’ll be on the phone within five minutes”.* Moreover, patients also highly valued the reminders and re-enforcing messages they receive from the telehealth team whenever they forgot to do their readings: “*The Telehealth staff, they always phone you to check that you have done your blood sugar; they remind you all the time…. Sometimes you tend to forget…”.*

#### Easy and convenient access to healthcare services

Easy access to the doctor and convenient health care services are important for the achievement of health equity and for improving everyone’s quality of life. This was evident during the interviews, where patients not only emphasised on the importance of having easy and convenient access to the doctor but also to a doctor that takes time for them and listens to them.

In this study, nearly all of the patients described their frustration of having experienced prolonged waiting times to speak/ see their GP or a hospital doctor, especially when it came to wanting to see a doctor of their choice as well. Their frustrations are evident in the following example statements:

“*I don’t dislike the doctors, but you just can’t get in to see them. If you’re on your own, you go say can I have an appointment and see them, and they say a week or whatever time. It doesn’t make a lot of sense, to my mind it don’t make a lot of sense….”.*

*“Telehealth is much easier. Whereas you’re phoning a doctor you’ve got to wait, God knows how long!”*

*“The last time I was sick I was waiting for the doctor until we couldn’t wait no more, we had to call the ambulance…”.*

Apart from describing the time concerns associated with receiving a doctor’s appointment, some patients also went on to describe that once seen by the doctor, they always seem too busy to care and listen to them, which makes them feel uneasy. One patient even described that she sometimes felt like she’s *“in their way”.* Others also described that they feel like they have no one to care for them apart from telehealth. One patient explained: *“Doctors are disgusting and you can’t get appointments….matrons very rarely come around…. I prefer Telehealth because they are there with me if I need them. And they will organize something or other if I need them…. And they will find out hospitals and ambulances and so forth…. So, at the moment they’re the only ones caring for me, do you understand? Well I feel it anyway…. I feel the only person that is looking out for me is Telehealth…. If I want to know something it’s like they’re all [doctors] leaving it up to you. Which is wrong…. I feel the doctors don’t want to know, they really don’t want to know….”.* Another patient even went as far as describing that it is probably because of her old age: “*I think in this country, they don’t care about the elderly… A lot of doctors now give up with all these patients…. They just don’t give that time…. We’ve got too many people in the country, they want to get rid of us, you know what I mean? Drop dead, should be easier…. And everybody I know of my age thinks the same... Because they don’t get the care, they don’t care about you, they don’t****…”.*** However, although the patient was disappointed at the limited care and support she has received so far from her doctor, she was extremely grateful for having telehealth by saying: *“Thank god I’ve got these people, these telehealth people… Gives me a bit of confidence there…”.*

Other patients also expressed their gratitude towards having telehealth and described that telehealth care services are more easily accessible than those received from their doctors: One patient noted: *“It’s quicker than seeing a doctor…. Quicker than getting help from one doctor sitting there stressed out!”.*

In addition to providing better access to care, patients and in particular those who were housebound or had mobility problems, also expressed their appreciation to telehealth for making care convenient for them by bringing health care and support into their homes, and thus, reducing their need for having to travel to doctor’s appointments. One patient described: *“They save us a lot of time for us to be going to the hospital. That’s a lot of time saving they have done for us. We don’t need to go to the doctor to do blood pressure and everything, or check our weight, as you will have a scale [at home] which can tell you exactly how much you weigh, then you go to the screen and see if you’ve put on weight or you’ve lost weight…”.* Two patients also added their concerns about the increased risk of contracting germs if they needed to travel to hospitals/ GP surgeries all the time and felt that it is much “safer” to be treated at home than physically having to visit their doctor: One of the patients described: *“Yes, it’s much better than keep popping into the doctor all the time. You sit down there for about an hour and a half and then you’re catching everybody else’s germs, all their colds and everything….”.*

#### Privacy concerns

Privacy challenges involved with using telehealth technologies could be of concern to some patients. However, only one patient in this study expressed a privacy concern with regards to not knowing who and how many people were monitoring him: *“I don’t like all my details like that for everybody to be monitoring. You don’t know who is at the other side….”.* None of the other patients expressed any concerns on this topic during the interviews.

#### Continuity of care

Majority of the patients in this study had an enthusiastic approach to the telehealth service and found it to help them stay healthy and out of hospital. However, a few concerns were highlighted during the interviews, which the patients felt could be improved when it comes to providing continuity of care with telehealth. These included the delivery of consistent quality of care, better communication between telehealth care members and more prompt home visits by matrons when patients are unwell. See the remarks from some of the patients:

*“The only thing that I do get a little bit peeved about is sometimes when they don’t get back to me. Sometimes, I wasn’t very well the other week, so I was in my bed and I didn’t do it [take readings], for about three or four days and they never checked me. I thought, they’re not checking so regularly as they used to...”.*

*“I can’t do the scales. That took a few times to sink in for them [telehealth team], why I wasn’t doing the weight. She went, “Oh.” She said, “I’ve read through the notes. Yes I can see it’s written down there.” So they’re obviously reading the notes. But then again, a couple of days after, someone else will phone up and ask me “why” and I say, “It’s in the notes! I know it, because one of your nurses told me”…. They can’t read the notes…. There is always a lack of communication between different characters…”.*

*“The only problem is the matrons and the doctors take too long to come out. The Telehealth team’s trying to do one thing, and the doctors and matrons are doing something totally different. That’s the problem. They’re not really working together, and when I do go bang, I do go bang! I could speak to Telehealth today; the matron will come in two or three days later. That’s the problem…”.*

Nevertheless, the above concerns did not affect the patients overall satisfaction for telehealth services. When all patients were asked for their overall perception of the telehealth care, patients replied with comments such as *“it’s very, very good”, “they’ve looked after me very well and allowed me to stay healthy despite of having diabetes”, “they monitor you very, very well, and for that I’m very grateful”, “this telehealth place is marvelous”, “I hate to be without it now”, “I would miss it very much if it weren’t here”, “I’ve got nothing but praise for it, I recommend it to many people”, “I think it’s marvelous, it’s better than having a doctor really”,* “*it’s a life saver*”, and “*I think that’s [telehealth/technology] the best thing that’s happened to me”.*

Although all of the patients were highly satisfied with the telehealth service, many patients viewed telehealth as a monitoring service only and is there to help them feel *“secure”.* One patient noted: “*To a certain extent telehealth is there for me but they’re not there to give me prescription and help me where I need it later wise….”.*

It was clear from the patients’ statements that they do not view telehealth as a complete substitute to normal care or face-to-face consultations. This is further elaborated in the below statements from some of patients, where they expressed the need for and their preference for seeing a doctor in person, in particular when it came to discussing more serious health concerns or the need for medical advice or physical examination:

*“It depends on what type of medical condition you have. You go to them and they like to examine you. For instance, like now I’m having terrible hip problems it’s difficult to walk, so you have to go and see a doctor face to face. I can’t sit on the phone and say, “Doctor, my hip.” They have to see me and find out what the problem is…”.*

*“You’re telling them your symptoms but they can’t actually see you so how can they really know what’s wrong with the person?”*

*“I prefer face to face when it comes to I’m not well, not with the Telehealth problem, no, that’s a different issue, but medically, yes, I would like a face to face on the condition. If like a flu or fever, I don’t mind, but when it comes to certain type of condition, yes, I do mind…”.*

Another patient also mentioned that the face-to-face consultation should preferable be with a doctor who they are familiar with: “*I know I can speak to Telehealth, but if it’s something that really, really concerns me, I’d phone my GP and speak to her. Because I know her face-to-face. With the Telehealth, you don’t…it’s just a voice at the end of a phone. And several different voices, it’s not just the one….”.*

### Theme 3: Empowerment

#### Patient education

All interviewed patients reported that they received interactive surveys, educational videos and motivational messages, tips, as well as questions tailored to their diagnoses on a daily basis from telehealth. When they were asked to comment on what their thoughts were on the information and material provided, a positive response was provided by all of the patients. They described the information to be educational and that it has helped them increase knowledge of their own condition(s), treatments and healthy living. One patient described: “*Videos that they put on, its quite educational videos about people that have diabetes and how they’re managing them at home… And also questionnaires about diabetes, the drugs and how you can manage it at home and things like that. So it’s quite educational in that sense…”.* Another patient noted: *“They give you little like, “Don’t eat before this time”, they give you all different tips. Did you know that? They say like, “Don’t forget to have this before your meal, or don’t forget to do this before this” …Lots of different tips on the telly [telehealth]…”.*

#### Supporting self-care with telehealth system’s health trend analysis

As soon as a patient transmits his/her readings via telehealth for clinical review, the data are immediately and automatically logged on the telehealth system. Patients can view the transmitted data on their television, which is displayed as a graph, providing an overall picture of the patient’s health over time. Patients can then follow the pattern of their health and well-being and see how it is being managed. The aim of this health trend analysis function is to help patients understand “the bigger” picture of their health, encourage them to self-reflect and take control of their own health. In this particular study, it was very evident during majority of the interviews that the health trend analysis function achieved what it was designed to do. One patient described: “*It helps me to be aware, yes, it gives you the trend, like sometimes when you’re doing the reading, there’s a line on the monitor that shows you the trend, the normal trend. And when it goes above that line it is higher than what it should be… you can see the graph yourself and it makes you feel like, yes, I have to eat the right thing, I have to do this and that. It gives you the motivation to do the right thing...”.*

Another patient also described that having to take her own readings at home with the telehealth equipment has not only increased her confidence in managing her own condition but it has also helped her come to terms with facing the reality of her conditions, which she describes, is a problem that many people with chronic conditions have: *“Because you’re doing it yourself, collecting the information and all of it. I mean, someone’s not coming along and sticking a bag on your arm every time, whatever. So it means that you are doing that yourself, and that’s nice because you can see yourself then if something’s wrong…so I think it has improved my confidence so far… It has also possibly helped me to accept the fact that I’ve got all these things. Because that’s another thing that people probably have a problem with it, it’s not the technology, it’s the fact they have to finally realise they’ve got very serious things wrong with them… well, it takes a while for you to come to terms with that. Particularly if you have had your life changed so much, like mine has….”.*

While taking and reviewing your own readings have influenced the patient above positively in terms of accepting and facing the reality of her health issues, this was not the case for another interviewed patient, who experienced panic attacks when she found out that her blood glucose levels were very high on her first day of using the telehealth equipment. She described that since “that moment of truth”, she has not used the telehealth again for monitoring her blood glucose because she knows her levels are still high and it scares her to have this confirmed using telehealth. However, she was happy to use telehealth for measuring her weight and blood pressure though because she knows those readings are within the normal range. In her own words: “*I haven’t used telehealth [for diabetes management] apart from the one day that she came and checked and put it on, I got scared…. I think if I knew that my sugar level was lower, I will just go on that machine continuously without being frightened…. with diabetes, I mean I’m really not confident but with the blood pressure and weight, yes. I’m quite excited about checking my weights and my blood pressure because I don’t really suffer with high blood pressure…”.*

Others who were not negatively affected by taking and reviewing their own readings also described that, because they know that the telehealth team will monitor their diabetic control through their transmitted readings, it encouraged them to be more careful when it comes to managing their diets. One patient described: “*It makes me more aware of what I’m doing. Sometimes you get a bit of slaphappy, you know what I mean? I like a bit of cake and I’ll have this, but because you’ve got to monitor yourself, you don’t have it. I think to myself, if I had that, it feels like cheating, you know what I mean? ……And when I do it on the telly [telehealth], they’re [telehealth team] going to know won’t they? There’s no hiding it…. Actually in that way, I think it’s quite good - It makes you think what you’re eating and all that”.*

## Discussion

### Summary of main findings

The findings from this interview study of 10 individuals with type 2 diabetes showed that telehealth is well received by all patients, many of who felt safer and better looked after. In addition, all patients reported that telehealth improved access to care and many also preferred telehealth care when compared with their regular face-to-face doctor’s appointments, though they would not want it to fully replace their contacts with their doctor, especially when it comes to discussing more serious health issues. This suggests that telehealth is only viewed as an additional health service to patients’ regular care and that it is important for them to still have access and communication with their doctor for specialist advice and continuity of care. This finding was echoed in Dario’s study exploring patients’ perceptions of telemedicine services [19].

### What this study adds to the literature

Our study expands the evidence base about the use of telehealth at home and support the growing consensus that telehealth can help provide more accessible care to patients [19–21]. It also add insight into the benefits and characteristics of telehealth that are important to people with type 2 diabetes, highlighting improved knowledge about their own health conditions and becoming more confident in self-management, as well as improved convenience, efficiency, quality of care and comfort. According to the WSD [22], it is claimed that telehealth can specifically help empower patients in becoming more knowledgeable about their own health conditions and improve self-management through the provision of self-monitoring tools. As a result, this could improve quality and more appropriate patient care as well as more effective use of healthcare resources as the need for hospital visits would be reduced for patients [23]. This claim is supported by the findings of this study. However, despite this and the convenience of managing their own health at home, this study, including research from the wider literature [24–26], have identified that there may be specific subgroups that are not suitable for telehealth, in particular those who suffer from anxiety/depression, low self-esteem, or have limited knowledge about their health condition(s). Hence, in order to ensure that people receive good continuity of care with telehealth, it is important to acknowledge and understand potential user behaviour and feelings toward technology first and whether it is feasible around their lifestyles before concluding that it is an acceptable mode of care for them.

### Implications for policy and practice

The positive findings associated with telehealth at home and type 2 diabetes may be useful to policy makers and practitioners who are re/designing or preparing to implement a similar telehealth system for patients with chronic condition(s) as it identifies patients’ perceptions of using technology to manage their own health at home before and after being introduced to telehealth as well as how and why patients benefitted from the telehealth system, including their views on what they believe could be further improved.

### Strengths and limitations

The main strength of this study was its exploratory nature and the use of a theoretical framework to guide the exploration, understanding and explanation of patients’ perceptions of using telehealth for type 2 diabetes management. The study reached saturation and the findings cohered with other telehealth studies available in the wider literature [24, 27–29]. The weaknesses were in relation to the sample. The sample included both patients new to telehealth as well as patients who have chosen to carry on using telehealth following the end of the larger WSD trial. During the interviews, patients were asked how long they have been using telehealth for and the duration ranged from 1.5 years to 3.5 years. This could potentially mean that majority of patients in the sample were post-WSD telehealth patients and therefore those who most certainly had a more favourable attitude towards telehealth even before their participation in the study. This could inevitable introduce bias into the sample and influence the direction of the results. In this case, it could potentially explain why there were limited negative views of the patients within the study. Moreover, as the study did not manage to recruit any of those who had withdrawn from a telehealth intervention, it is very possible that the patient sample of this study is skewed towards those with a more optimistic view towards the technology and is therefore not an appropriate representation of the entire patient population of telehealth. Nevertheless, this study provides insights of the potential impact telehealth may have on patients with type 2 diabetes.

## Conclusion

The findings of the study demonstrate the feasibility of telehealth monitoring at home as well its potential benefits in people living with type 2 diabetes. Overall, the evidence from this study showed that telehealth has the potential to enhance patient’s quality of life, allow them to live independently at home as well as help them take and be in more control over their own health state. It therefore supports the use of telehealth for the routine care of people with type 2 diabetes.

However, although findings from this study are promising, one must interpret them with caution due to limitations identified in the sample. Future research should attempt to include people from the wider telehealth user population, including healthcare professionals who are one of the main users, to ensure that the findings do not disadvantage any unrepresented groups as well as to establish all the possible factors that facilitate or hinder the uptake of telehealth services.

## Abbreviations

COPD: Chronic obstructive pulmonary disease
NHS: National Health Service
WSD: Whole system demonstrator
